# Development of Burdock Root Inulin/Chitosan Blend Films Containing Oregano and Thyme Essential Oils

**DOI:** 10.3390/ijms19010131

**Published:** 2018-01-03

**Authors:** Thi Luyen Cao, So-Young Yang, Kyung Bin Song

**Affiliations:** Department of Food Science and Technology, Chungnam National University, Daejeon 34134, Korea; ctluyenagu@gmail.com (T.L.C.); sygrim@gmail.com (S.-Y.Y.)

**Keywords:** burdock root, chitosan, edible film, essential oil, inulin

## Abstract

In this study, inulin (INU) extracted from burdock root was utilized as a new film base material and combined with chitosan (CHI) to prepare composite films. Oregano and thyme essential oils (OT) were incorporated into the INU-CHI film to confer the films with bioactivities. The physical and optical properties as well as antioxidant and antimicrobial activities of the films were evaluated. INU film alone showed poor physical properties. In contrast, the compatibility of INU and CHI demonstrated by the changes in attenuated total reflectance-Fourier transformation infrared spectrum of the INU-CHI film increased tensile strength and elongation at break of the INU film by 8.2- and 3.9-fold, respectively. In addition, water vapor permeability, water solubility, and moisture content of the films decreased proportionally with increasing OT concentration in the INU-CHI film. Incorporation of OT also increased the opacity of a and b values and decreased the L value of the INU-CHI films. All INU-CHI films containing OT exhibited antioxidant and antimicrobial properties. Particularly, the INU-CHI film with 2.0% OT exhibited the highest 2,2′-azino-bis(3-ethylbenzothiazoline-6-sulphonic acid), 2,2-diphenyl-1-picrylhydrazyl radical scavenging, and antimicrobial activities against four pathogens. Thus, the INU-CHI film containing OT developed in this study might be utilized as an active packaging material in the food industry.

## 1. Introduction

Inulin (INU) is a polysaccharide with a β-d-fructose chain linked by (2 → 1) glycosidic bonds and one terminal α-d-glucopyranose molecule bound to a fructose chain through a (1 ↔ 2) glycosidic linkage. INU is mainly found in artichoke tubers, chicory, yacon, and burdock roots. Because of its physicochemical properties, INU has been used in various foods as a versatile modifier to replace fat or sugar, enhance stability and spreadability, and improve the organoleptic and physical properties of food products [1]. The degree of polymerization (DP) of INU is 2–60 depending on the plant source, maturity at harvest, post-harvest conditions, and processing techniques [2]. It has been reported that high-DP INU molecules exhibit microcrystal-forming and gel-forming capacities and compatibility with other polymers [3]. Based on the physicochemical characteristics of INU, such as its film-forming capacity, it can be used with other polymers to prepare blend films. However, INU extracted from burdock roots has not been studied as an edible packaging material.

Chitosan (CHI) is a hetero-polymer with 2-amino-d-glucose and 2-acetamido-d-glucose monomers linked through β-1,4-glycosidic linkages [4,5]. CHI is a popular biomaterial for edible film and coating production [6]. Because of its biocompatibility, antimicrobial activities, low oxygen permeability, and good mechanical properties, CHI has been combined with other hydrocolloids such as starch, gelatin, and alginate to produce blended or multi-layered films [7,8,9].

Antimicrobial and/or antioxidant substances have been incorporated into active packaging films [10]. Oregano essential oil (OE) and thyme essential oil (TE) are two of the most commonly used essential oils for the preparation of edible films because of their antimicrobial and antioxidant activities [11]. Carvacrol, thymol, γ-terpinene, and *p*-cymene are the main bioactive compounds found in OE and TE. Carvacrol is a major component in OE, while thymol is the most abundant in TE [12,13]. A combination of OE and TE or carvacrol and thymol has been reported to show synergistic or additive effects on antimicrobial activity [14,15].

Therefore, we developed blended films of INU extracted from burdock root with CHI. In addition, to confer antioxidant and antimicrobial activities, OE and TE were incorporated into the blend films and the physical and optical properties and antioxidant and antimicrobial activities of the developed films were characterized.

## 2. Results and Discussion

### 2.1. Characterization of Inulin (INU) Extracted from Burdock Root

**Figure 1:**
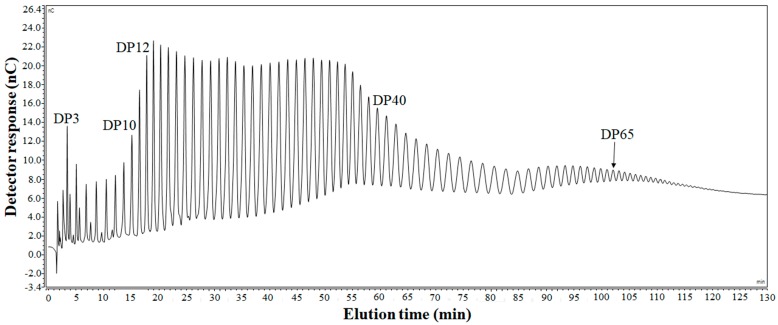
Ion chromatogram of inulin (INU) extracted from burdock root.

The extracted INU was approximately 9% on a dry weight basis of burdock root and the moisture content of the INU extract was 9.6%. Figure 1 illustrates the DP profile of the extracted INU, and each peak represents a specific DP. The DP of INU extracted from burdock root ranged from 3 to 65. The relative area of oligosaccharides with DP ranging from 1 to 10 was too small, accounting for 6.0% of the peak area. In contrast, INU molecules with DP values from 11 to 40 were more predominant, accounting for 74.7% of the total peak area. In addition, there was a gradual decrease in the peak area of INU with DP values of 41–65. The results of DP distribution can be explained by the high solubility of oligosaccharides with DP lower than 10 in 70% ethanol as a solvent. As a result, these oligosaccharides were somewhat decanted with the supernatant during centrifugation, whereas most INU molecules with DP higher than 10 were precipitated in the solvent during INU extraction [16].

### 2.2. Physical Properties of the Films

Table 1 shows the tensile strength (TS) and elongation at break (E) of the prepared INU films. INU showed film-forming capacity in the presence of sorbitol as a plasticizer. However, the TS of the INU film was considerably lower than that of CHI film. The low TS of the INU film is likely due to the low DP of the extracted INU molecules and flexibility of the INU polymer. Mensink et al. [2] reported that INU contains furanose rings that contribute to polymer flexibility. In addition, unlike the pyranose ring structure of amylose, the furanose rings are not in the line of the backbone of INU molecules, making the INU molecules mobile and less rigid. In contrast, the CHI films showed high TS and low E [17], and the INU-CHI film had good TS (26.58 MPa) and E (19.98%) values. Similar results were reported when CHI was blended with starch film [18].

The incorporation of oregano and thyme essential oils (OT) decreased TS and increased E. Particularly, the TS of INU-CHI films containing 1.0% and 2.0% OT decreased to 23.33 and 17.08 MPa, respectively, whereas the E values of the films increased to 23.08% and 25.42%, respectively. These changes in TS and E may be attributed to OT disrupting the film matrix. The loose structure of the film matrix by the addition of OT decreased TS. In contrast, OT can behave as a plasticizer, which favors stretching of the polymer and increases E. Similar results were obtained when OE was incorporated into cassava starch-chitosan films [19] and TE was added to CHI films [17].

The water vapor permeability (WVP), water solubility (WS), and moisture content (MC) of the films are presented in Table 2. The WVP and WS of the INU film were higher than those of CHI film. The higher WVP and WS of the INU film may also be because of the larger number of hydroxyl groups and lower DP of INU molecules compared to on CHI molecules. INU exhibited strong water absorption capacity because of the formation of hydrogen bonds with water molecules. The hydrophilicity of the INU molecules increased with decreasing DP [2,20]. Thus, the combination of CHI and INU decreased the hydrophilic properties of the INU film. Bangyekan et al. [21] reported a similar result where the increase in surface hydrophobicity of the starch film coated with CHI was proportional to CHI content. The hydrophobic properties of the CHI film are mainly related to the presence of acetyl groups in the CHI molecules [6]. Particularly, the MC of the INU film was 11.32%, much lower than that of the CHI film (21.59%). These results may be related to the less compact structure of the INU film produced by short and flexible chains of INU. The loose network of the INU film demonstrated by the low TS is related to poor water retention capacity, and water molecules easily evaporated from the INU matrix during drying of the films.

The incorporation of OT in the INU-CHI film decreased WVP, WS, and MC of the films. The increase in hydrophobicity of the INU-CHI films was principally ascribed to the presence of phenolic compounds, particularly carvacrol and thymol in OT. The decreased WVP with the addition of TE into the CHI film was also reported by Perdones et al. [22]. In addition, there was a negative correlation between OT concentration and WVP, WS, and MC of the films. Similarly, the water vapor barrier of the CHI film was enhanced by incorporating carvacrol into the CHI film [23], and increased proportionally with increasing oregano concentration [19].

### 2.3. Optical Properties

Opacity contributes to the light barrier capacity of the films. The results showed that INU film was opaque compared to the transparent CHI film (Table 3). The opacity of the INU film was 21.90 A/mm, whereas that of CHI film was only 0.38 A/mm. The combination of INU and CHI decreased the inherent transparency of the CHI film. The opacity of INU-CHI film was 3.28 A/mm. A similar result was reported when corn starch was combined with CHI to form a blended film [24]. Furthermore, opacity of the film increased when OT was incorporated into the INU-CHI film and proportionally increased with increasing OT concentration. The increase in opacity with the addition of OT may be related to the presence of oil globules in the film matrix, causing light scattering at the interface of OT [25]. Similarly, it was reported that the opacity of the gelatin-chitosan composite film increased with increasing OE concentration [26].

INU film had light brownish-yellow color because of the presence of phenolic compounds in burdock roots, which are involved in browning reactions during the slicing and drying of burdock root [27]. Subsequently, incorporation of OT decreased the L (lightness) value and increased both the a (redness) and b (yellowness) values of the INU-CHI films. The change in colors of the INU-CHI films containing OT was because of the brownish-yellow color of the OT mixture. Similarly, there were a decrease in the L value and increases in the a, b, and opacity values of the CHI films incorporated with TE [28]. Perdones et al. [22] also reported that an increasing TE concentration significantly decreased the L value of the CHI film.

### 2.4. Attenuated Total Reflectance-Fourier Transformation Infrared (ATR-FTIR) Spectroscopy

**Figure 2:**
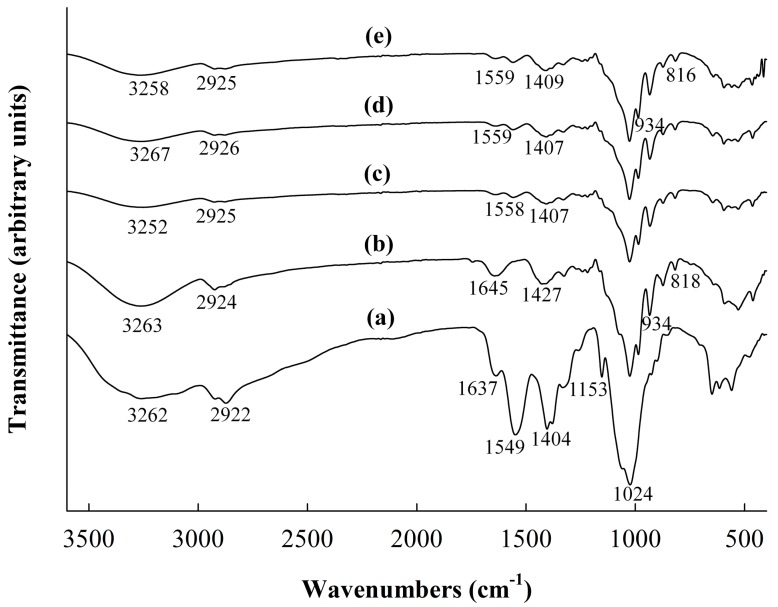
The Attenuated Total Reflectance-Fourier Transformation Infrared (ATR-FTIR) spectra of the films. (**a**) CHI film, (**b**) INU film, (**c**) INU-CHI film, (**d**) INU-CHI-OT 1.0 film, (**e**) INU-CHI-OT 2.0 film.

The ATR-FTIR was used to examine the molecular properties of the INU and CHI films and determine the possible interactions between the blend films. Figure 2 shows the spectra of the films. The overall spectrum of the INU film had similar characteristics to that of pure INU determined in the previous studies [29,30]. Particularly, the peak at 3263 cm^−1^ was assigned as an –OH group and hydrogen bond stretching vibrations (SV), and the peaks at 2924 and 1645 cm^−1^ were attributed to the C–H and C=O SV, respectively [31]. The presence of the ether bonds (C–O–C) was indicated by a sharp peak at 1025 cm^−1^, whereas that of fructose molecules with β-glycosidic linkages was characterized by peaks at 934, 873, and 818 cm^−1^ [29,30]. For CHI film, the broad peak from 3100 to 3500 cm^−1^ was due to O–H overlap with N–H SV [10]. The peaks at 2922 and 2873 cm^−1^ indicated C–H stretching in –CH_2_ and –CH_3_ groups, respectively [32]. The SV of amide I and N–H was determined at 1637 and 1549 cm^−1^, respectively [10]. The peaks at 1404 and 1379 cm^−1^ also corresponded to the C–H in –CH_3_ and –CH_2_ groups, respectively [32,33]. The sharp peak at 1024 cm^−1^ was also responsible for the SV of the C–O in C–O–C group [10].

In the spectrum of the INU-CHI film (Figure 2c), there were decreases in the intensity at 3100–3500 cm^−1^ for all INU-CHI films with or without OT. In addition, the peaks at 3262–3263 cm^−1^, representing –OH SV in the INU and CHI films, were shifted to 3252 cm^−1^ in the INU-CHI films. These changes suggest interactions between –OH groups of INU and –NH_2_ or –OH of CHI by hydrogen bonds. Furthermore, the compatibility between INU and CHI was confirmed by the absence of a peak at 1645 cm^−1^ in the INU film and a shift of the peak at 1549 cm^−1^ (assigned for N–H SV) in the CHI spectrum to 1558 cm^−1^ in the INU-CHI spectrum. The interactions of the two polymers may eventually change the physical properties of the INU-CHI film. Similarly, the interactions between corn starch and CHI in the composite film were reported [10]. In contrast, the incorporation of OE and TE into the INU-CHI film did not change the spectra of the INU-CHI films containing OT compared to the INU-CHI film without OT. This may be because of evaporation of OT under the vacuum condition during FTIR operation.

### 2.5. Scanning Electron Microscopy Images of the Inulin (INU), Chitosan (CHI), and INU-CHI Films

**Figure 3:**
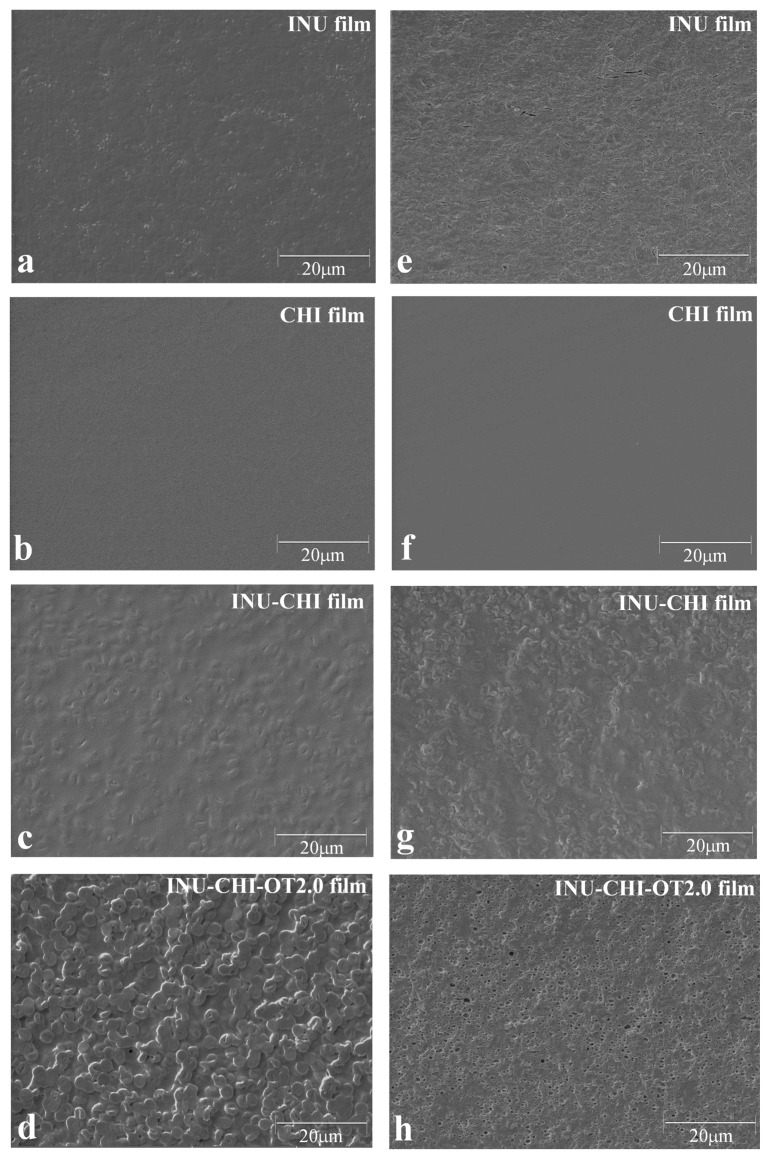
Surface scanning electron microscopy (SEM) images of the (**a**) INU film; (**b**) CHI film; (**c**) INU-CHI film; (**d**) INU-CHI-OT 2.0 film. Cross-sectional SEM images of the (**e**) INU film; (**f**) CHI film; (**g**) INU-CHI film; (**h**) INU-CHI-OT 2.0 film. Magnification: 3000× for surface and cross-section.

The microstructures of the surface and cross-section of the INU, CHI, and INU-CHI films are shown in Figure 3. The surface of the CHI film was smooth, whereas those of films containing INU were rough. The rough surface of the INU and INU-CHI films was mainly because of the presence of INU molecules, which were rearranged and aggregated in the solution, resulting in the formation of spherulites [34]. Aggregation of INU molecules may occur during film drying. The spherical INU crystals distributed in the INU-CHI films caused a decrease in the continuity of the film matrix. Similarly, the distribution of INU with DP higher than 23 in the caprine milk cheese matrices caused the interruption of casein-fat networks, resulting in less stiff and non-cohesive structures [35]. Similar to the appearance of the surface structure, the cross-section of the INU film exhibited a fragile structure with the presence of small cracks, whereas that of the CHI film was compact and homogenous. These results clearly explain the low TS and E of the INU film as well as high cohesiveness of the CHI film. In addition, the microstructure of the INU film is consistent with its high WVP and WS.

The addition of essential oils increased the heterogeneity of the films, with rough surfaces and pores observed in the cross-section of INU-CHI films containing OT. Particularly, the INU-CHI-OT 2.0 film showed the roughest surface and most discontinuous image. Pores formed because of the self-association of hydrophobic OT molecules in the hydrophilic matrix during drying of the films. Similar results for the increase in heterogeneity of the CHI films by the incorporation of essential oils were reported [28,36].

### 2.6. Antioxidant Activities of Films Containing Oregano and Thyme Essential Oils (OT)

**Figure 4:**
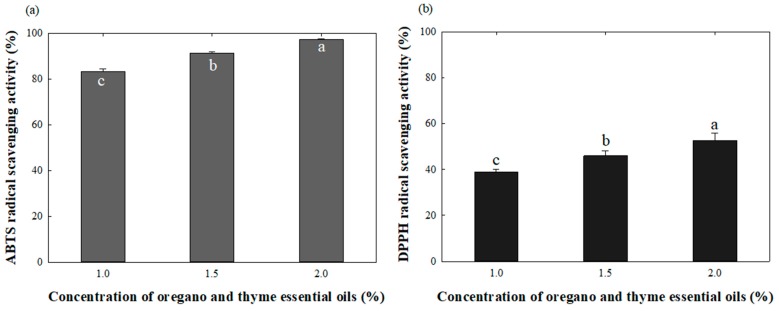
Antioxidant activities of the INU-CHI films containing OT. (**a**) 2,2′-azino-bis(3-ethylbenzothiazoline-6-sulphonic acid) (ABTS) radical scavenging activity; (**b**) 2,2-diphenyl-1-picrylhydrazyl (DPPH) radical scavenging activity. ^a–c^: Any means with different letters are significantly (*p* < 0.05) different by Duncan’s multiple range test.

Antioxidant activities of the films were evaluated based on 2,2′-azino-bis(3-ethylbenzothiazoline-6-sulphonic acid) (ABTS) and 2,2-diphenyl-1-picrylhydrazyl (DPPH) radical scavenging activities. Compared to the INU-CHI film without OT, the INU-CHI film containing 1.0% OT showed 83.29% and 38.79% ABTS and DPPH radical scavenging activities, respectively (Figure 4). The antioxidant properties of the films were attributed to bioactive compounds of OT present in the film matrix. Carvacrol and thymol were determined to be the main components with strong antioxidant activities of OE and TE [15,37]. The antioxidant capacities of carvacrol and thymol were conferred by their hydroxyl groups donating protons [38]. In addition, the antioxidant activities of the films were proportional to the OT content. Similarly, the carvacrol-incorporated gelatin films exhibited increasing antioxidant activities in accordance with carvacrol concentration [39]. In addition, the DPPH radical scavenging activity of the quince seed mucilage films containing OE increased proportionally with increasing OE content [37].

### 2.7. Antimicrobial Activities of Films Containing OT

Table 4 shows the antimicrobial activities of the films against four pathogens, *Listeria monocytogenes*, *Staphylococcus aureus*, *Escherichia coli*, and *Salmonella typhimurium*. The INU-CHI film without OT showed no antimicrobial activity against the pathogens. In contrast, all INU-CHI films containing OT showed antimicrobial activities against the tested pathogenic bacteria. Antimicrobial activity against the bacteria is mainly ascribed to carvacrol and thymol, which disrupt the bacterial cell membrane, inhibit microbial enzymes, and disturb intracellular metabolic pathways [40]. The inhibition of INU-CHI films containing OT against *L. monocytogenes* and *S. aureus* was considerably more effective than that against *E. coli* and *S. typhimurium*. This is mainly because of the difference in the cell wall structures of the two bacterial groups, Gram-positive and Gram-negative bacteria. Gram-negative bacteria have an external lipopolysaccharide membrane surrounding the cell wall, which exhibits barrier capacity against hydrophobic essential oil molecules [40]. These results are in good agreement with those of other reports, where OE and TE were less effective against Gram-negative than Gram-positive bacteria [15,41]. Among the four tested pathogens, *S. typhimurium* was the most resistant bacterium to OT. This result is consistent with that of Pelissari et al. [19]. As expected, the antimicrobial activities of the INU-CHI films with OT proportionally increased with increasing OT concentration.

## 3. Materials and Methods

### 3.1. Materials

Burdock (*Arctium lappa*) root was provided by Korea Nongkeum Association (Daejeon, Korea). CHI with deacetylation of 75–85% was purchased from Showa Chemical Industry Co. (Tokyo, Japan). OE was obtained from dōTERRA (Pleasant Grove, UT, USA). TE was purchased from Gooworl Co. (Daegu, Korea). Tween 80, sorbitol, and glycerol were obtained from Sigma-Aldrich (St. Louis, MO, USA). Glacial acetic acid (99.8% purity) was purchased from Samchun Company (Daejeon, Korea).

### 3.2. Extraction of INU

Burdock root was washed, sliced, dried at 65 °C for 24 h, and ground. First, INU extraction was carried out by dispersing burdock powder in distilled water (1:20, *w*/*v*) at 90 °C and stirring the suspension at 900 rpm for 15 min. Next, the suspension was homogenized at 11,000 rpm for 5 min, sonicated at 25 °C for 10 min, sieved, and centrifuged at 10,000× *g*, 25 °C for 15 min to remove insoluble materials. Second, the extracted INU was precipitated in ethanol (70%) at 4 °C for 24 h. Finally, INU was obtained by centrifuging the slurry at 10,000× *g*, 4 °C for 15 min, drying the precipitates at 45 °C for 12 h, and pulverizing INU into fine powder.

### 3.3. Determination of DP of INU Extracted from Burdock Roots

A Dionex ICS-5000+ ion chromatography system (Thermo Fisher Scientific, Waltham, MA, USA) with an electrochemical detector was used to determine the DP of INU extracted from burdock root. INU was dissolved in 10% dimethyl sulfoxide, and the dissolved INU was injected into a DionexTM CarboPacTM PA1 column at a flow rate of 1 mL/min and eluted with the varying gradients of 0.6 M sodium acetate in 0.15 M NaOH as 10–30% for 0–10 min, 40–60% for 10–52 min, and 65–85% for 52–132 min.

### 3.4. Film Preparation

CHI (2.5%, *w*/*v*) film-forming solution (FFS) was prepared by stirring CHI powder in 1% acetic acid solution at 180 rpm for 8 h. Glycerol (20% of CHI, *w*/*w*) was added to the solution as a plasticizer. Based on preliminary experiments for the preparation of INU FFS, INU (4%, *w*/*v*) and sorbitol (40% of INU, *w*/*w*) were mixed and stirred in distilled water at 40 °C, 400 rpm for 30 min. The INU-CHI FFS was prepared by mixing CHI and INU FFS at a ratio of 1:1. In addition, INU-CHI films containing OT were prepared by incorporating varying amounts (1%, 1.5%, and 2%) of OT (OE:TE, 1:1, *w*/*w*) into the FFS. Tween 80 (10% of OT, *w*/*w*) was used as a surfactant in films containing OT. All FFS were homogenized at 10,000 rpm for 5 min, sonicated at 25 °C, filtered through 2 layers of gauze, and degassed for 5 min. Next, prepared solutions were casted onto the petri dish (9 cm in diameter) and dried at 25 °C, relative humidity (RH) of 50 ± 3% for 14 h to produce the INU film, CHI film, INU-CHI film, and INU-CHI films containing OT (INU-CHI-OT 1.0, INU-CHI-OT 1.5, and INU-CHI-OT 2.0 for the INU-CHI films with 1.0%, 1.5%, and 2.0% OT, respectively). The obtained films were stored at 25 °C, RH 50 ± 3% for 24 h before characterization.

### 3.5. Physical Properties Measurement

TS and E of the films were determined as described by Cao et al. [42]. WVP of the film was determined three times at 25 °C and a RH of 50% as described by Lee et al. [43]. Five square dry films (4 cm^2^) of each sample were used to determine WS of the films. The films were dipped in 20 mL of distilled water and slightly shaken at 25 °C for 24 h. The WS was defined as the percentage of film weight loss compared to the initial weight of the film. MC of the films was determined by drying five square films (2 × 2 cm) of each sample at 105 °C for 24 h. The MC was calculated as described by Cao et al. [42]. The experiment was conducted in triplicate.

### 3.6. Optical Properties of the Films

A colorimeter (Minolta, CR-400, Tokyo, Japan) was used to measure Hunter values (L, a, and b) of the films. Five film sheets (2 × 2 cm) of each sample were measured by placing them on a standard plate having L = 97.18, a = −0.02, and b = 1.99. Opacity of the film was calculated based on Equation (1). At least five film sheets (2 × 2 cm) were used to measure absorbance at 600 nm using a UV-VIS spectrophotometer (UV-2450, Shimadzu Corporation, Kyoto, Japan).

(1) $$\mathrm{Opacity}=\frac{A_{b}}{T}$$

where *A*_b_ is the absorbance of the film at 600 nm and *T* is the thickness of the film (mm).

### 3.7. ATR-FTIR Spectroscopy

ATR-FTIR spectra of the INU, CHI, and INU-CHI films with or without OT were determined using Bruker Vertex 80v Vacuum Infrared Spectrometer (Billerica, MA, USA) with Deuterated Triglycine Sulphate detector. Analyses were performed with diamond/ZnSe crystals at 2 cm^−1^ spectral resolution over a wavenumber range of 400–4000 cm^−1^, with 16 scans acquired for each measurement.

### 3.8. Scanning Electron Microscopy

A scanning electron microscope (LYRA3 XMU; Tescan, Warrendale, PA, USA) was used to obtain microscopic images of the surface and cross-section of the films at an accelerating voltage of 5.0 kV and magnification of 3000×.

### 3.9. Antioxidant Activities

The two free radical scavenging assays, ABTS and DPPH, were used to determine the antioxidant activities of the films. The film extract solutions were prepared by shaking the films (0.1 g, 0.3 × 0.3 cm) in 9.9 mL distilled water at 37 °C, 150 rpm for 30 min, followed by centrifugation at 3000× *g* for 10 min to discard undissolved materials. The ABTS assay was performed as described by Lee et al. [43]. The DPPH assay was conducted as described by Yang et al. [44].

### 3.10. Antimicrobial Activities

The antimicrobial activities of the films containing OT with different concentrations against the four common pathogens were determined using the disc diffusion assay described by Kim et al. [45]. *L. monocytogenes* (KCTC 13064, ATCC 15313), *S. aureus* (KCTC 1621, ATCC 10537), *E. coli* O157:H7 (ATCC 43889, NCTC 12079), and *S. typhimurium* (ATCC 14028, KCTC 2421) inocula at concentrations of 6–7 log CFU/mL were spread onto the surface of Mueller Hinton Agar media. Next, paper discs (8 mm in diameter) impregnated with 80 μL of FFS were placed onto the inoculated Mueller Hinton Agar discs. The diameters of inhibition zones were measured after incubation of the discs at 37 °C for 24 h.

### 3.11. Statistical Analysis

The SAS program (SAS Institute, Cary, NC, USA) was used to analyze the experimental data using analysis of variance and Duncan’s multiple range tests (*p* < 0.05). The results were expressed as the mean ± standard deviation.

## 4. Conclusions

In this study, INU extracted from burdock root was used as a new biomaterial for edible film preparation. The INU film exhibited poor physical properties, such as low TS and E and high WVP and WS. In contrast, the incorporation of CHI enhanced the physical properties of the INU film. The compatibility of INU and CHI in the blend film was confirmed by the ATR-FTIR spectrum of the INU-CHI film. In addition, the INU-CHI film containing OT showed strong antioxidant activities. The INU-CHI films containing OT also showed antimicrobial activities against pathogens. Therefore, the developed INU-CHI films containing OT can be used as an antioxidant and antimicrobial edible film.

## Figures and Tables

**Table 1:** Physical properties of the inulin (INU), chitosan (CHI), and INU-CHI films containing oregano and thyme essential oils (OT).

Samples	Tensile Strength (MPa)	Elongation at Break (%)
INU	5.10 ± 0.38 ^f^	5.11 ± 0.45 ^d^
CHI	41.65 ± 1.76 ^a^	5.22 ± 0.32 ^d^
INU-CHI	26.58 ± 1.44 ^b^	19.98 ± 1.26 ^c^
INU-CHI-OT 1.0	23.33 ± 1.92 ^c^	23.08 ±1.73 ^b^
INU-CHI-OT 1.5	20.02 ± 0.98 ^d^	25.71 ± 1.83 ^a^
INU-CHI-OT 2.0	17.08 ± 0.76 ^e^	25.42 ± 2.80 ^a^

Means ± S.D. ^a–f^: any means in the same column followed by different letters are significantly (*p* < 0.05) different by Duncan’s multiple range test.

**Table 2:** Water vapor permeability, water solubility, and moisture content of the INU, CHI, and INU-CHI films containing OT.

Essential Oil Concentration (%)	Water Vapor Permeability (10^−9^ g/m s Pa)	Water Solubility (%)	Moisture Content (%)
INU	4.23 ± 0.19 ^a^	62.12 ± 1.40 ^a^	11.32 ± 0.45 ^f^
CHI	3.48 ± 0.09 ^c^	22.39 ± 0.62 ^e^	21.59 ± 0.31 ^a^
INU-CHI	3.74 ± 0.27 ^b^	35.15 ± 0.46 ^b^	17.38 ± 0.29 ^b^
INU-CHI-OT1.0	3.46 ± 0.17 ^c^	34.94 ± 0.57 ^b^	15.73 ± 0.29 ^c^
INU-CHI-OT1.5	3.21 ± 0.15 ^d^	33.12 ± 0.89 ^c^	14.08 ± 0.23 ^d^
INU-CHI-OT2.0	3.15 ± 0.19 ^d^	32.41 ± 0.93 ^d^	12.23 ± 0.16 ^e^

Means ± S.D. ^a–f^: any means in the same column followed by different letters are significantly (*p* < 0.05) different by Duncan’s multiple range test.

**Table 3:** Optical properties of the INU, CHI and INU-CHI films containing OT.

Samples	Opacity (A/mm)	L	a	b
INU	21.90 ± 0.36 ^a^	79.34 ± 0.47 ^f^	−0.04 ± 0.01 ^a^	23.65 ± 0.41 ^a^
CHI	0.38 ± 0.03 ^f^	96.59 ± 0.16 ^a^	−2.99 ± 0.20 ^e^	11.49 ± 0.39 ^e^
INU-CHI	3.28 ± 0.05 ^e^	89.51 ± 0.80 ^b^	−2.08 ± 0.03 ^d^	18.39 ± 1.25 ^d^
INU-CHI-OT1.0	3.66 ± 0.04 ^d^	86.55 ± 0.42 ^c^	−1.95 ± 0.04 ^c^	20.36 ± 0.80 ^c^
INU-CHI-OT1.5	4.00 ± 0.06 ^c^	85.64 ± 0.45 ^d^	−1.94 ± 0.02 ^c^	20.75 ± 0.54 ^bc^
INU-CHI-OT2.0	4.70 ± 0.12 ^b^	84.87 ± 0.36 ^e^	−1.82 ± 0.01 ^b^	21.02 ± 0.57 ^b^

Means ± S.D. ^a–f^: any means in the same column followed by different letters are significantly (*p* < 0.05) different by Duncan’s multiple range test.

**Table 4:** Antimicrobial activities of the INU-CHI films containing OT against pathogens.

	Inhibition Zone (mm)			
Essential Oil Concentration (%)	*L. monocytogenes*	*S. aureus*	*E. coli* O157:H7	*S. typhimurium*
INU-CHI	ND *	ND	ND	ND
INU-CHI-OT1.0	16.50 ± 0.32 ^c^	17.07± 0.17 ^c^	12.82 ± 0.29 ^c^	11.80 ± 0.18 ^c^
INU-CHI-OT1.5	17.31 ± 0.18 ^b^	18.14 ± 0.19 ^b^	16.38 ± 0.23 ^b^	12.57 ± 0.19 ^b^
INU-CHI-OT2.0	19.38 ± 0.17 ^a^	18.95 ± 0.18 ^a^	18.44 ± 0.28 ^a^	14.64 ± 0.12 ^a^

Means ± S.D. ^a–c^: any means in the same column followed by different letters are significantly (*p* < 0.05) different by Duncan’s multiple range test. * ND: Not detected.
